# Elevated plasma bile acids coincide with cardiac stress and inflammation in young *Cyp2c70*^−/−^ mice

**DOI:** 10.1038/s41390-024-03596-4

**Published:** 2024-10-02

**Authors:** Hilde D. de Vries, Tim R. Eijgenraam, Vincent W. Bloks, Niels L. Mulder, Tim van Zutphen, Herman H. W. Silljé, Folkert Kuipers, Jan Freark de Boer

**Affiliations:** 1 Department of Laboratory Medicine, University of Groningen, University Medical Center Groningen, Groningen, The Netherlands; 2 Faculty Campus Fryslân, University of Groningen, Leeuwarden, The Netherlands; 3 Department of Pediatrics, University of Groningen, University Medical Center Groningen, Groningen, The Netherlands; 4 Department of Cardiology, University of Groningen, University Medical Center Groningen, Groningen, The Netherlands; 5 European Research Institute for the Biology of Ageing (ERIBA), University of Groningen, University Medical Center Groningen, Groningen, The Netherlands

## Abstract

**Background:**

High plasma bile acids (BAs), for instance due to intrahepatic cholestasis of pregnancy or neonatal cholestasis, are associated with cardiac abnormalities. Here, we exploited the variability in plasma BA levels in *Cyp2c70*^−/−^ mice with a human-like BA composition to investigate the acute effects of elevated circulating BAs on the heart.

**Methods:**

RNA sequencing was performed on hearts of 3-week-old *Cyp2c70*^−/−^ mice lacking mouse-specific BA species that show features of neonatal cholestasis. Cardiac transcriptomes were compared between wild-type pups, *Cyp2c70*^−/−^ pups with low or high plasma BAs, and *Cyp2c70*^−/−^ pups from dams that were perinatally treated with ursodeoxycholic acid (UDCA).

**Results:**

We identified 1355 genes that were differentially expressed in hearts of *Cyp2c70*^−/−^ mice with high versus low plasma BAs with enrichment of inflammatory processes. Strikingly, expression of 1053 (78%) of those genes was normalized in hearts of pups of UDCA-treated dams. Moreover, 645 cardiac genes strongly correlated to plasma BAs, of which 172 genes were associated with cardiovascular disease.

**Conclusions:**

Elevated plasma BAs alter gene expression profiles of hearts of mice with a human-like BA profile, revealing cardiac stress and inflammation. Our findings support the notion that high plasma BAs induce cardiac complications in early life.

**Impact:**

- *Cyp2c70*^−/−^ mice with a human-like bile acid composition show features of neonatal cholestasis but the extrahepatic consequences hereof have so far hardly been addressed
- Elevated plasma bile acids in *Cyp2c70*^−/−^ pups coincide with cardiac stress and inflammation
- Perinatal treatment with UDCA prevents dysregulated cardiac gene expression patterns in *Cyp2c70*^−/−^ pups

## Introduction

Liver disease and heart disease often coincide.^1^ Particularly, cholestatic liver disorders, which are characterized by high plasma levels of bile acids (BAs), have been associated with cardiac abnormalities.^2^ For example, intrahepatic cholestasis of pregnancy (ICP) has been reported to cause cardiac arrhythmias and cardiac arrest in the unborn child.^3^ As such, N-terminal prohormone of brain natriuretic peptide (NT-proBNP), a gold standard plasma biomarker in the diagnosis of heart failure,^4^ was found to be increased in fetuses from ICP pregnancies.^5,6^ Moreover, hereditary cholestatic liver diseases often progress to liver cirrhosis.^7^ Up to 50% of adult patients with liver cirrhosis develop cirrhotic cardiomyopathy, a disorder that strongly affects cardiac functionality.^8^ Therefore, endocrine crosstalk via BAs has been implicated as a possible link between liver and heart disease.^2,3^

BAs exert hormonal functions through activation of BA receptors.^9^ Various transmembrane BA receptors, such as muscarinic receptors^10^ and G protein-coupled bile acid receptor 1 (GPBAR1, also known as Takeda G protein-coupled receptor 5 [TGR5]),^10–13^ as well as nuclear BA receptors, including farnesoid X receptor (FXR)^14^ and vitamin D receptor (VDR),^15^ have been reported to be present in the heart. In addition, in vitro studies have shown that taurocholic acid (TCA) reduced the contraction rate and calcium transient amplitude in isolated rat cardiomyocytes.^16,17^ Taken together, these findings provide a rationale to study the role of circulating BAs and cardiac complications.

Experimental animal research frequently involves mouse models of human diseases. Yet, the abundant presence of mouse-specific muricholic acids (MCAs), which possess atypical signaling characteristics, limits translation of preclinical studies that focus on (patho)physiological BA actions to the human situation.^18^ Following the discovery that the CYP2C70 enzyme is responsible for the synthesis of MCAs, we and others have generated *Cyp2c70*-deficient mice.^19–21^ We reported that 3-week-old *Cyp2c70*^−/−^ pups with a human-like BA composition exhibited features of neonatal cholestasis, characterized by elevated BAs and liver transaminases in plasma, which could be prevented by perinatal treatment of dams with ursodeoxycholic acid (UDCA).^20^ Thus, *Cyp2c70*-deficient mice represent a valuable model to investigate BA-related liver diseases with improved translatability.

Here, we employed *Cyp2c70*^−/−^ mouse pups as an in vivo model system for cardiac exposure to high concentrations of human-like BAs, and exploited the natural variation in circulating plasma BA levels in the mice at the age of 3 weeks to gain insight into the molecular alterations that are involved in the early phase of BA-induced cardiomyopathy. We performed RNA sequencing on hearts of *Cyp2c70*-deficient pups, and we correlated transcriptional changes to plasma BA levels. Additionally, we investigated the effect of perinatal UDCA exposure on cardiac mRNA expression patterns in *Cyp2c70*^−/−^ pups.

## Materials and methods

### Experimental animals and study design

We used 3-week-old *Cyp2c70*^−/−^ pups (C57BL/6J-Cyp2c70^em3Umcg^)^20^ as a model to explore the effects of elevated plasma BA concentrations on the heart. Breeding was performed with 10- to 18-week-old *Cyp2c70*^+/−^ males and *Cyp2c70*^+/−^ females (heterozygous x heterozygous), resulting in wild-type (WT, *Cyp2c70*^+/+^), *Cyp2c70*^+/−^ and *Cyp2c70*^−/−^ offspring. To prevent the onset of cholestatic features in *Cyp2c70*^−/−^ pups, a part of the *Cyp2c70*^+/−^ dams received chow supplemented with 0.1% UDCA (w/w) (Sigma-Aldrich, MO) during breeding, gestation, and suckling. Both male and female offspring were included since we had not observed overt phenotypic sex differences at the age of 3 weeks in our earlier studies.^20,22^ WT offspring of UDCA-fed dams and *Cyp2c70*^+/−^ offspring were not included in this study. At 3 weeks of age, pups were euthanized, and blood and organs were collected as described below. All animal experiments were executed in accordance with the Dutch law and were approved by the Dutch Central Committee for Animal Experiments and the Animal Welfare Body of the University of Groningen (permit numbers AVD105002015244 and IVD15244-02-051).

### Euthanasia and tissue collection

Mice were anesthetized with isoflurane, and were euthanized by exsanguination due to blood collection, followed by cervical dislocation. Hearts were excised, washed with saline, and weighed, after which atria were removed. Hearts and livers were snap-frozen in liquid nitrogen and stored at −80 °C for transcriptomics. Due to the small size of the pups’ hearts, we used sections of hearts obtained in a previous study for histology,^22^ in which the entire hearts of 3-week-old *Cyp2c70*^−/−^ pups were flushed with saline and fixed in formalin.

### Plasma parameters

BAs in plasma were measured by ultra-high-performance liquid chromatography-tandem mass spectrometry (UHPLC-MS/MS) on a Nexera X2 UHPLC system (Shimadzu, Japan) coupled to a QTRAP 4500MD triple quadrupole mass spectrometer (SCIEX, MA), and were quantified using stable isotopically labeled internal standards as reported previously.^23^ Plasma levels of alanine aminotransferase (ALT), aspartate aminotransferase (AST), alkaline phosphatase (ALP) and total bilirubin were quantified using a cobas 6000 analyzer with standard reagents (Roche Diagnostics, the Netherlands).

### Real-time quantitative polymerase chain reaction

Snap-frozen hearts were thoroughly ground and homogenized, and total RNA was isolated using an RNeasy Fibrous Tissue Mini Kit (Qiagen, Germany). Next, cDNA synthesis was performed using the QuantiTect RT kit (Qiagen) according to the manufacturer’s instructions. Gene expression was determined on a CFX384 Touch real-time PCR detection system (Bio-Rad, CA) using iQ SYBR green supermix (Bio-Rad) and custom-designed primers (*Nppa*: 5′-GCTTCCAGGCCATATTGGAG-3′ and 5′-GGTGGTCTAGCAGGTTCTTG-3′; *Rplp0*: 5′-AAGCGCGTCCTGGCATTGTC-3′ and 5′-GCAGCCGCAAATGCAGATGG-3′) as published before.^24^ Ct values were determined with CFX Manager software (version 3.0) (Bio-Rad), normalized to housekeeping gene *Rplp0* (ribosomal protein lateral stalk subunit P0, also known as 36B4), and displayed relative to the WT group.

### Transcriptomic analysis

RNA sequencing was performed by Novogene (China) using a HiSeq Sequencing system and 150 bp paired-end technology (Illumina, CA). Reads were aligned to the mouse genome (version GRCm38/mm10), and gene counts were determined with featureCounts.^25^ Principal component analysis (PCA) and differential gene expression analysis were performed with the *DESeq2* package (version 1.42.0)^26^ in R (version 4.3.2). Gene set enrichment analysis (GSEA) was performed with the *fgsea* package (version 1.28.0)^27^ in R using the mouse hallmark and Reactome gene sets (version 2023.2.Mm) of the Molecular Signatures Database.^28^ Differential expression and gene set enrichment with a Benjamini-Hochberg-adjusted *p*-value < 0.05 were considered significant.

### Cardiac collagen deposition

Formalin-fixed, paraffin-embedded cardiac tissues of 3-week-old *Cyp2c70*^−/−^ pups of our prior work^22^ were cut into 4 μm thick sections. Masson’s trichrome staining was performed to detect collagen deposition as a measure of fibrosis using routine procedures.^29^ Photographic images were obtained using a Nanozoomer 2.0-HT slide scanner (Hamamatsu Photonics, Japan), and the percentage of myocardial fibrosis in the entire stained section was quantified using the Positive Pixel Count v9 algorithm of Aperio’s ImageScope software (version 12.4.6) (Leica Biosystems, Germany).

### Statistical analyses

For statistical analyses other than for RNA sequencing data, multigroup comparisons were done with Kruskal-Wallis tests, followed by Conover post hoc tests using BrightStat online software.^30^ Data are plotted as Tukey box-and-whisker plots unless noted otherwise. For correlations, skewed data (according to the Shapiro-Wilk test) were log-transformed, and Pearson’s correlation coefficients were determined. *P*-values < 0.05 were considered significant.

## Results

### *Cyp2c70*^−/−^ pups show cholestatic features with elevated plasma BAs and liver injury markers

**Fig. 1:**
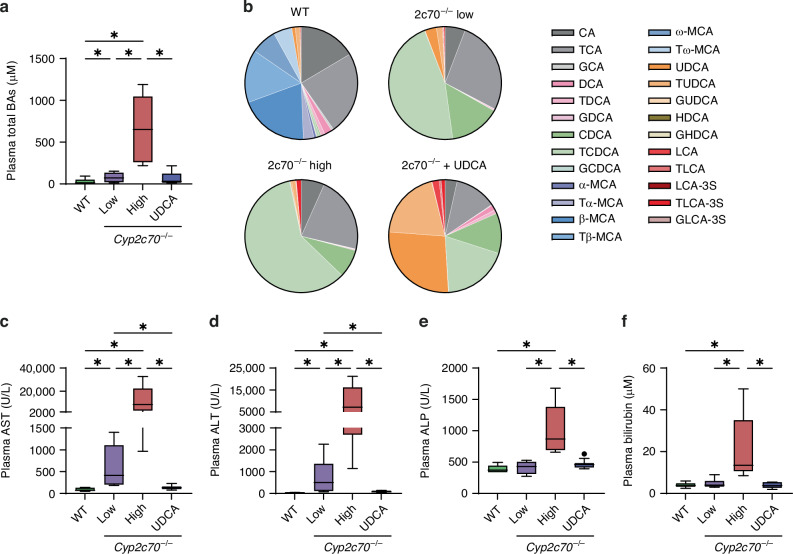
Three-week-old *Cyp2c70*^−/−^ pups demonstrate features of cholestasis. Total BA concentrations (**a**), BA composition (**b**) and AST (**c**), ALT (**d**), ALP (**e**) and bilirubin (**f**) levels in plasma of WT pups (*n* = 6), *Cyp2c70*^−/−^ pups with low or moderately elevated plasma BAs (‘2c70^−/−^ low’, *n* = 6), *Cyp2c70*^−/−^ pups with high plasma BAs (‘2c70^−/−^ high’, *n* = 5) and *Cyp2c70*^−/−^ pups of which dams were perinatally administered UDCA (*n* = 12). *P  <  0.05 (Kruskal-Wallis test followed by Conover post hoc comparisons). 2c70, *Cyp2c70*; ALP, alkaline phosphatase; ALT, alanine aminotransferase; AST, aspartate aminotransferase; BA, bile acid; UDCA, ursodeoxycholic acid; WT, wild type.

To investigate the impact of high concentrations of circulating BAs on the heart, we studied 3-week-old *Cyp2c70*^−/−^ pups with a hydrophobic human-like BA composition and features of neonatal cholestasis. In line with previous publications,^20,22^ plasma BA levels were significantly increased in 3-week-old *Cyp2c70*^−/−^ pups compared to their WT littermates, which could be prevented by perinatal exposure to UDCA (Supplementary Fig. 1a). A marked heterogeneity in plasma BA concentrations was apparent, which allowed us to assess the effect of variable BA levels in the circulation on the heart. To this aim, we divided *Cyp2c70*^−/−^ pups into a group with normal or moderately elevated BA concentrations ( < 200 µM, ‘2c70^−/−^ low’) and a group that displayed a strong cholemic phenotype ( ≥ 200 µM, ‘2c70^−/−^ high’) (Fig. 1a).

Since BAs possess species-specific properties, i.e. potency for BA receptor stimulation or cytotoxicity, we determined the BA composition in plasma of WT and *Cyp2c70*^−/−^ pups. As expected, the majority of BAs in plasma of WT pups consisted of MCAs (51.6%), whereas MCAs were not detected in plasma of *Cyp2c70*^−/−^ pups (Fig. 1b). Instead, chenodeoxycholic acids (CDCAs) were highly abundant in plasma of *Cyp2c70*^−/−^ pups. No differences were observed between plasma BA composition of 2c70^−/−^ high pups versus 2c70^−/−^ low pups (Fig. 1b, Supplementary Fig. 1b). In *Cyp2c70*^−/−^ pups of which dams perinatally received UDCA, the average percentage of UDCAs in plasma was 47.2% of total BAs, indicating that UDCA was successfully incorporated into the pup’s BA pool (Fig. 1b). No differences in body, liver or heart weight were found between groups (Supplementary Fig. 1c–e). Yet, 2c70^−/−^ low pups had significantly increased plasma levels of liver damage markers AST and ALT compared to WT pups (Fig. 1c–d). Moreover, 2c70^−/−^ high pups displayed increased levels of AST, ALT, ALP, and total bilirubin as compared to WT and 2c70^−/−^ low pups (Fig. 1c–f). Together, these results confirmed the presence of features of cholestasis and liver damage in these 3-week-old pups.

### Circulating BA levels correlate with cardiac ANP expression in *Cyp2c70*^−/−^ pups

**Fig. 2:**
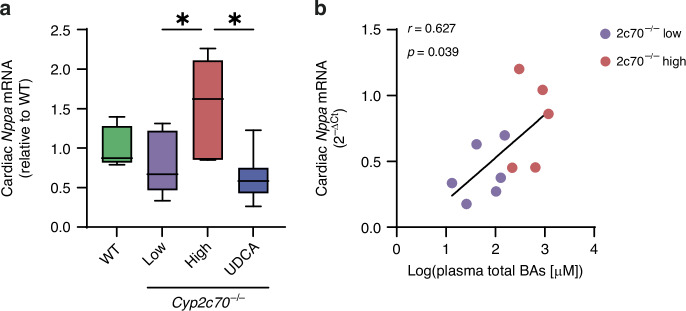
Cardiac *Nppa* expression is increased in 3-week-old *Cyp2c70*^−/−^ pups with high plasma BA concentrations. **a** Cardiac mRNA levels of *Nppa* in WT pups (*n* = 6), *Cyp2c70*^−/−^ pups with low or moderately elevated plasma BAs (‘2c70^−/−^ low’, *n* = 6), *Cyp2c70*^−/−^ pups with high plasma BAs (‘2c70^−/−^ high’, *n* = 5) and *Cyp2c70*^−/−^ pups of which dams were perinatally administered UDCA (*n* = 12). *Nppa* was normalized to the housekeeping gene *Rplp0* (36B4). Data are shown relative to the WT group. *P  <  0.05 (Kruskal-Wallis test followed by Conover post hoc comparisons). **b** Correlation between plasma total BA concentrations (log-transformed) and cardiac *Nppa* gene expression (2^−ΔCt^) in 2c70^−/−^ low and 2c70^−/−^ high pups. Pearson’s correlation coefficient (*r*) and *p*-value are shown. 2c70, *Cyp2c70*; BA, bile acid; *Nppa*, natriuretic peptide A; UDCA, ursodeoxycholic acid; WT, wild type.

Next, we measured ventricular gene expression of natriuretic peptide A (ANP, *Nppa*) to assess whether there was cardiac stress in *Cyp2c70*^−/−^ pups. No significant differences were observed in cardiac *Nppa* expression between WT and 2c70^−/−^ low pups (Fig. 2a), suggesting that the human-like BA composition in *Cyp2c70*^−/−^ pups in itself did not result in cardiac stress. In contrast, 2c70^−/−^ high pups demonstrated significantly increased cardiac *Nppa* expression compared to 2c70^−/−^ low pups, indicating that the elevated concentrations of human-like BAs did cause stress to the heart of these pups (Fig. 2a). Myocardial *Nppa* levels were comparable between *Cyp2c70*^−/−^ pups that had been perinatally exposed to UDCA and WT and 2c70^−/−^ low pups (Fig. 2a), demonstrating that cardiomyocyte stress was prevented by UDCA treatment. Moreover, plasma BA concentrations significantly positively correlated (*r* = 0.627, *p* = 0.039) with cardiac *Nppa* expression in *Cyp2c70*^−/−^ pups (Fig. 2b).

### The cardiac transcriptome reveals active inflammation in *Cyp2c70*^−/−^ pups with high plasma BAs

**Fig. 3:**
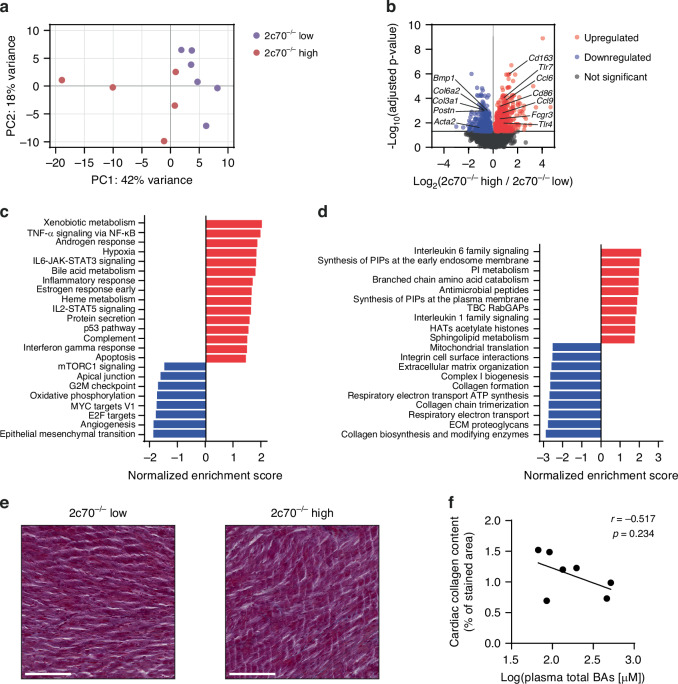
Marked alterations in the cardiac transcriptome of 3-week-old *Cyp2c70*^−/−^ pups with high plasma BAs. **a** PCA plot of cardiac gene expression profiles of *Cyp2c70*^−/−^ pups with low or moderately elevated plasma BAs (‘2c70^−/−^ low’, *n* = 6) and *Cyp2c70*^−/−^ pups with high plasma BAs (‘2c70^−/−^ high’, *n* = 5). **b** Volcano plot of differential gene expression between *Cyp2c70*^−/−^ pups with high plasma BAs (‘2c70^−/−^ high’) versus *Cyp2c70*^−/−^ pups with low or moderately elevated plasma BAs (‘2c70^−/−^ low’). The horizontal line corresponds to an adjusted *p*-value of 0.05. Significantly enriched hallmark gene sets (**c**) and top ten most positively and negatively enriched Reactome pathways (**d**) as determined from the differential expression data in (**b**). **e** Masson’s trichrome-stained heart sections of *Cyp2c70*^−/−^ pups with low or moderately elevated plasma BAs (‘2c70^−/−^ low’) and *Cyp2c70*^−/−^ pups with high plasma BAs (‘2c70^−/−^ high’). Scale bars represent 100 μm. **f** Correlation between plasma total BAs (log-transformed) and collagen deposition in hearts of *Cyp2c70*^−/−^ pups from our earlier work.^22^ Pearson’s correlation coefficient (*r*) and *p*-value are shown. 2c70, *Cyp2c70*; BA, bile acid; PC, principal component.

The observed increase in cardiac ANP expression prompted us to perform RNA-seq on hearts of *Cyp2c70*^−/−^ mice to evaluate the molecular alterations that are caused by elevated plasma BA levels. PCA revealed that the cardiac gene expression profile of 2c70^−/−^ high pups was different than the cardiac transcriptome of 2c70^−/−^ low pups (Fig. 3a). WT pups, 2c70^−/−^ low pups and *Cyp2c70*^−/−^ pups that had been perinatally exposed to UDCA clustered together (Supplementary Fig. 2). Except for sex-specific expression of chromosomal genes, there were no apparent sex differences. We found 1355 significantly (adjusted *p*-value < 0.05) differentially expressed genes (DEGs), of which 819 were upregulated and 536 were downregulated in 2c70^−/−^ high pups (Fig. 3b). As shown in Fig. 3c, functional enrichment analysis using the mouse hallmark gene sets of the Molecular Signatures Database revealed that the DEGs were positively enriched for terms that are related to the immune system, including ‘TNF-α signaling via NF-κB’, ‘IL6-JAK-STAT3 signaling’ and ‘inflammatory response’, indicating activation of inflammatory pathways in the heart upon exposure to high plasma BA concentrations. Consistently, immune-related pathways ‘interleukin 1 family signaling’ and ‘interleukin 6 family signaling’ were among the top ten most positively enriched Reactome pathways (Fig. 3d). Other positively enriched hallmark gene sets included ‘hypoxia’ and ‘apoptosis’, suggesting an activated stress response, as well as ‘bile acid metabolism’, which was enriched by the differential expression of BA-related genes such as *Cyp27a1*, *Cyp39a1* and *Nr0b2* (Supplementary Table 1), which implies activation of BA signaling in the heart. The most negatively enriched hallmark gene set was ‘epithelial-mesenchymal transition’ (EMT) (normalized enrichment score −1.94, adjusted *p*-value 1.39e-06), which is a process that normally occurs during development and has been associated with cardiac fibrosis.^31^ Correspondingly, the negative enrichment score of this pathway resulted from the downregulation of, amongst others, *Bmp1*, *Postn*, *Loxl1* and several collagen genes (including *Col6a2*, *Col3a1* and *Col1a2*), and therefore seemed to be driven by genes involved in extracellular matrix (ECM) assembly (Supplementary Table 1).^32^ Downregulation of ECM genes was also reflected by the top 10 most negatively enriched Reactome pathways, which included ‘collagen biosynthesis and modifying enzymes’, ‘collagen chain trimerization’, ‘collagen formation’ and ‘extracellular matrix organization’ (Fig. 3d, Supplementary Table 2).

To assess whether transcriptional changes of collagens and ECM turnover genes resulted in altered collagen deposition in the heart, we performed the Masson’s trichrome staining on cardiac tissues of 3-week-old *Cyp2c70*^−/−^ pups from a previously published cohort,^22^ and we quantified the collagen content (Fig. 3e, f). No significant correlation between myocardial collagen deposition and plasma BA concentrations was observed in 3-week-old Cyp2c70^−/−^ pups (*r* = −0.517, *p* = 0.234).

### Perinatal UDCA treatment of dams prevents differential cardiac gene expression in *Cyp2c70*^−/−^ pups

**Fig. 4:**
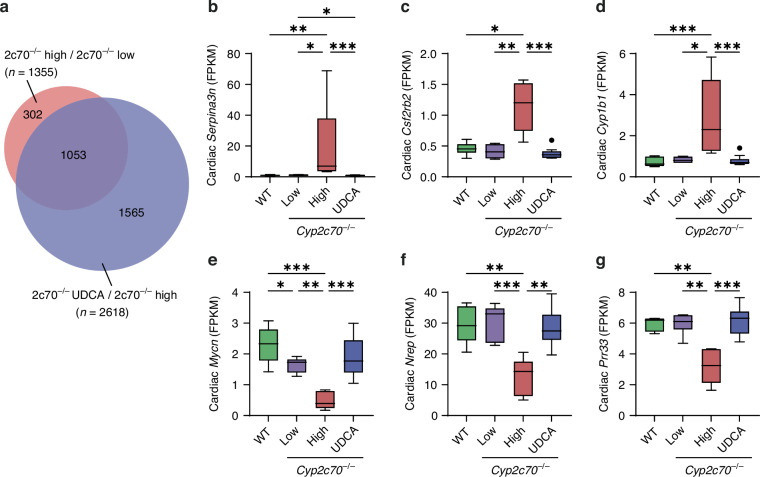
Cardiac gene expression is normalized in *Cyp2c70*^−/−^ pups upon perinatal UDCA treatment of dams. **a** Venn diagram showing the overlap (purple) between differentially expressed genes in *Cyp2c70*^−/−^ pups with high plasma BAs (‘2c70^−/−^ high’) versus *Cyp2c70*^−/−^ pups with low or moderately elevated plasma BAs (‘2c70^−/−^ low’) (as shown in Fig. 3b) (red circle) and in *Cyp2c70*^−/−^ pups of which dams were perinatally administered UDCA (*n* = 12) versus *Cyp2c70*^−/−^ pups with high plasma BAs (‘2c70^−/−^ high’, *n* = 5) (blue circle). Normalized transcript counts (FPKM) of genes that were most significantly upregulated (*Serpina3n*, *Csf2rb2* and *Cyp1b1*) (**b**–**d**) or most significantly downregulated (*Mycn*, *Nrep* and *Prr33*) (**e**–**g**) in 2c70^−/−^ high pups as compared to 2c70^−/−^ low pups and were normalized in 2c70^−/−^ + UDCA pups. A brief description of the genes that are displayed here is provided in Supplementary Table 3. *P < 0.05 (Kruskal-Wallis test followed by Conover post hoc comparisons). 2c70, *Cyp2c70*; BA, bile acid; *Csf2rb2*, colony stimulating factor 2 receptor, beta 2; *Cyp1b1*, cytochrome P450, family 1, subfamily b, member 1; *Mycn*, N-myc proto-oncogene; *Nrep*, neuronal regeneration related protein; *Prr33*, proline rich 33; *Serpina3n*, serine peptidase inhibitor, clade A, member 3 N; UDCA, ursodeoxycholic acid; WT, wild type.

Since UDCA is commonly prescribed to ICP patients, we evaluated whether perinatal UDCA administration of dams could prevent the observed differential cardiac gene expression patterns in *Cyp2c70*^−/−^ pups. We found 2618 DEGs (1100 upregulated and 1518 downregulated) in hearts of *Cyp2c70*^−/−^ pups of which the mothers had been given a UDCA-supplemented diet during gestation and suckling as compared to the untreated 2c70^−/−^ high pups. Comparison of these genes to the DEGs that were identified comparing 2c70^−/−^ high pups to 2c70^−/−^ low pups revealed an overlap of 1053 genes (78%) (Fig. 4a). More detailed evaluation demonstrated that all 1,053 genes were upregulated in hearts of 2c70^−/−^ high pups and were downregulated in UDCA-treated *Cyp2c70*^−/−^ pups or vice versa (Fig. 4b–g, Supplementary Table 3).

### Cardiovascular disease-associated genes correlate with plasma BA levels in *Cyp2c70*^−/−^ pups

**Fig. 5:**
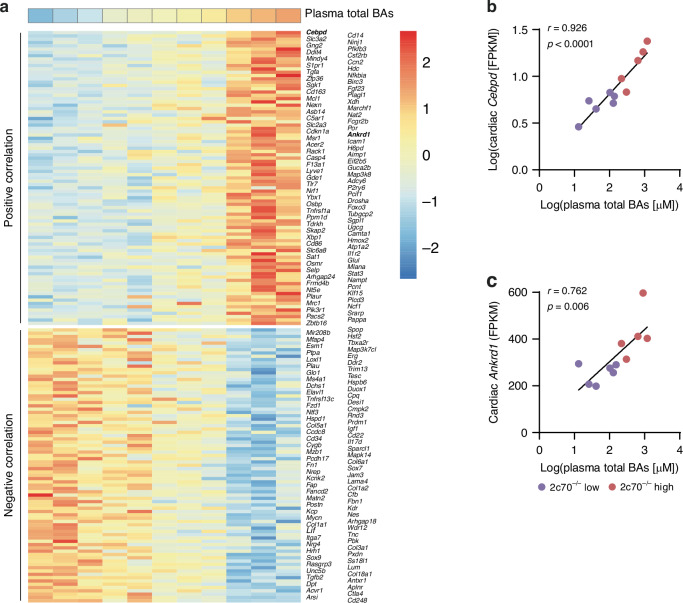
Genes that have been associated with cardiovascular diseases correlate to plasma BA concentrations in *Cyp2c70*^−/−^ pups. **a** Heatmap of cardiac genes (rows) that strongly correlated (*p* < 0.01) to plasma BA concentrations in *Cyp2c70*^−/*-*^ pups with low or moderately elevated plasma BAs (‘2c70^−/−^ low’) and *Cyp2c70*^−/−^ pups with high plasma BAs (‘2c70^−/−^ high’) (columns, sorted from left to right by plasma total BAs). Correlation between plasma total BA concentrations (log-transformed) and normalized transcript counts (FPKM) of cardiac *Cebpd* (**b**) or cardiac *Ankrd1* (**c**) in *Cyp2c70*^−/*−*^ pups with low or moderately elevated plasma BAs (‘2c70^−/−^ low’) and *Cyp2c70*^−/−^ pups with high plasma BAs (‘2c70^−/−^ high’). Pearson’s correlation coefficient (*r*) and *p*-value are shown. 2c70, *Cyp2c70*; *Ankrd1*, ankyrin repeat domain 1; BA, bile acid; *Cebpd*, CCAAT enhancer binding protein delta.

To gain more insight into which genes in the heart are directly linked to the exposure to circulating BAs, we correlated the normalized gene counts (fragments per kilobase million [FPKM]) of each individual gene to plasma BA levels in *Cyp2c70*^−/−^ pups. A total of 645 genes strongly (*p* < 0.01) correlated with plasma BA concentrations, of which 307 were positively correlated and 338 were negatively correlated. Finally, we explored the potential functional implications of these BA-correlated genes using the DisGeNET discovery platform, which records gene-disease associations from numerous sources.^33^ We found that 172 genes that we identified as directly correlating with plasma BA levels have been shown to associate with cardiovascular diseases (Fig. 5). Taken together, these findings implicate that high BA concentrations in the blood early in life can lead to differential gene expression patterns in the neonatal heart, corresponding to transcriptional changes that are known to occur in cardiovascular diseases.

## Discussion

In this study, we demonstrate that cardiac expression of ANP, a well-established marker for heart disease, was increased and positively correlated with plasma BA levels in *Cyp2c70*-deficient pups with high circulating BA levels, indicating that hypercholanemia induces stress in the heart. Analysis of the cardiac transcriptome revealed activation of inflammatory pathways and disturbed ECM homeostasis in *Cyp2c70*^−/−^ pups with high plasma BAs. Moreover, numerous cardiovascular disease-related genes strongly correlated to total BA concentrations in plasma. Finally, we found that perinatal UDCA treatment prevented hypercholanemia and the majority of the observed myocardial alterations.

In accordance with our previous reports,^20,22^ the 3-week-old *Cyp2c70*-deficient pups used in the current study had a human-like BA composition that is devoid of MCAs, and display features of neonatal cholestasis. As such, *Cyp2c70*^−/−^ mice had increased plasma cholestasis markers, including liver transaminases, ALP, total bilirubin, and, importantly, elevated plasma BA concentrations compared to WT littermates. Furthermore, the variable plasma BA levels in *Cyp2c70*^−/−^ mice enabled us to explore concentration-dependent effects of circulating BAs. Altogether, these features make *Cyp2c70*^−/−^ mice a valuable model to study the impact of BAs on extrahepatic tissues like the heart.

Elevated plasma BA levels were associated with a transcriptional profile that indicated cardiac stress and inflammation in hearts of *Cyp2c70*^−/−^ mice. First, using a targeted approach, we found that ANP expression was induced in hearts of *Cyp2c70*^−/−^ mice with high plasma BAs. In fact, cardiac ANP levels significantly positively correlated with plasma total BAs. Natriuretic peptides, including ANP and BNP, are synthetized and secreted by cardiomyocytes under pathologic conditions,^34^ indicating activation of a stress response in the hearts of cholestatic *Cyp2c70*^−/−^ mice. Secondly, the unbiased transcriptomic approach revealed the induction of a hypoxic stress response and apoptotic signaling, as well as upregulation of various immune system-related genes in the hearts of *Cyp2c70*^−/−^ mice with high plasma BAs. Conceivably, the inflammatory pathways are upregulated in response to BA-induced injury to the heart. Cardiac injury triggers a reparative response that consists of an acute, transient inflammation, followed by proliferative and reparative programs.^35^ During the inflammatory phase, cardiomyocytes secrete pro-inflammatory stimuli CC chemokine ligand (CCL) 2, interleukin (IL)-1, IL-6, and tumor necrosis factor (TNF) to attract monocytes, neutrophils, and leukocytes.^35^ Consistently, we observed a positive enrichment for IL1 and IL6 signaling and upregulation of several chemokines, such as *Ccl6* and *Ccl9*, in the hearts of *Cyp2c70*^−/−^ mice with high plasma BAs. In addition, a greater abundance of immune cells was evidenced by higher mRNA levels of cluster of differentiation (CD) markers and receptors that are expressed by immune cells, including *Cd163*, *Cd86*, toll-like receptors *Tlr7* and *Tlr4*, and *Fcgr3* (Fig. 3b). Following the inflammation phase, fibroblasts are activated and differentiate into myofibroblasts to promote myocardial remodeling through deposition and cross-linking of collagens and other ECM proteins to preserve the structural integrity of the heart.^36^ However, we did not observe induction of an expression profile in *Cyp2c70*^−/−^ mice with high plasma BAs that corresponds to tissue remodeling and fibrosis formation. In contrast, myofibroblast marker α-smooth muscle actin (*Acta2*), several collagen genes and multiple enzymes involved in ECM turnover were downregulated. Yet, the myocardial collagen content as determined by histology did not differ significantly between *Cyp2c70*^−/−^ mice with high versus low plasma BAs. Considering the young age of the mice, it is conceivable that we captured the early inflammatory phase in our transcriptomic analyses, while the hearts had yet not advanced to the reparative stage in the 3-week-old mice. Taken together, these findings implicate that high plasma BA levels coincided with a stress response as well as inflammation in the hearts of 3-week-old *Cyp2c70*^−/−^ mice, which, possibly due to the young age of the mice, had not progressed to fibrosis formation.

For many years, it has been recognized that BAs exert hormone-like functions besides their classic role as intestinal ‘soaps’ to aid the digestion of lipids,^9^ which enables them to also exert effects in tissues that are not involved in the enterohepatic circulation of BAs. Accordingly, in our current study, GSEA revealed ‘bile acid metabolism’ as one of the most positively enriched processes in the hearts of *Cyp2c70*^−/−^ mice with high plasma BAs. Among the genes that contributed to the enrichment score of this gene set was *Nr0b2*, encoding the small heterodimer partner (SHP) protein. As SHP is the downstream target of the nuclear BA receptor FXR, induction of *Nr0b2* suggests activation of FXR signaling. Indeed, expression of FXR in the heart has been reported before,^14^ and was also observed in our dataset. Furthermore, transcripts of several BA transporters (*Slc10a1*, *Slc10a6*, *Slco1a4* and *Slco2b1*) were present in our data, potentially allowing active uptake of circulating BAs into cardiomyocytes. Indeed, Pu et al.^14^ reported that cardiomyocyte-expressed FXR mediates disruption of mitochondrial integrity and cardiomyocyte apoptosis. Similarly, there was also negative enrichment of mitochondrial genes in hearts of *Cyp2c70*^−/−^ mice with high plasma BAs, suggesting disturbed mitochondria. Noteworthily, Mao et al. recently found upregulation of the BA synthesis genes *Cyp27a1*, *Cyp7b1* and *Hsd3b7* in carnitine acetyltransferase (CRAT)-deficient neonatal rat ventricular cardiomyocytes (NRVMs).^37^ The authors speculated that synthesis of BAs in cardiomyocytes may have cytotoxic effects to the heart. However, in the current study, we did not find indications that this pathway is active in *Cyp2c70*^−/−^ mice with high plasma BA concentrations (data not shown), indicating that the observed cardiac alterations are caused by high BA levels in the circulation.

We found that the expression of 645 cardiac genes strongly correlated to plasma total BA levels in *Cyp2c70*^−/−^ pups, of which 172 genes are associated with cardiovascular disease. The strongest correlation (*r* = 0.926) was observed for *Cebpd*, encoding CCAAT/enhancer-binding protein (C/EBP) delta (Fig. 5a, b). C/EBPs have been shown to increase upon myocardial ischemia, and inhibition of C/EBP signaling preserved cardiac function post-infarction.^38^ Similarly, ankyrin repeat domain 1 (*Ankrd1*) was among the genes in hearts of *Cyp2c70*^−/−^ mice which most strongly correlated with plasma BAs (Fig. 5c). Multiple studies have demonstrated that *Ankrd1* expression is increased in failing hearts, and that cardiac overexpression of *Ankrd1* induced cardiomyopathy or exacerbated myocardial dysfunction following myocardial infarction or pressure overload in mice.^39,40^ Together, these results further support the notion that elevated BAs in the systemic circulation can contribute to heart disease.

Pregnant women that are diagnosed with ICP are often treated with UDCA. While UDCA has been proven to relieve symptoms in the mother, benefits for the fetus remain unconfirmed.^41,42^ Here, we show that perinatal administration of UDCA to dams prevented the majority of the altered expression profile in hearts of *Cyp2c70*^−/−^ pups, demonstrating that UDCA treatment could inhibit BA-induced myocardial alterations. Whether the beneficial effects of UDCA are the result of incorporation of UDCA into the BA pool (i.e. a direct effect of UDCA on the heart) or the consequence of lowered total plasma BAs (i.e. an indirect effect via the liver), is yet unknown. UDCA may protect cardiomyocytes from BA-induced alterations via its anti-inflammatory effects, anti-apoptotic effects and/or via reducing endoplasmic reticulum (ER) stress. However, further studies are required to elucidate the direct mechanisms of UDCA on the heart.

Some limitations of our study should be taken into account. Although the observed transcriptional alterations in the hearts of *Cyp2c70*^−/−^ pups indicate cardiac stress and inflammation, we did not assess the functional impact of neonatal cholestasis on cardiomyocyte survival or contractility. As our study was performed in 3-week-old mice, progression towards tissue repair was not observed on the transcriptional level, and one could therefore argue that functional impairment is unlikely to be apparent at this age. Other groups have reported rhythm disturbances in isolated cardiomyocytes incubated with TCA^16,17^ or cardiac hypertrophy in adult FXR/SHP double knockout mice.^43^ The findings of the latter study may be different from the results obtained in our mouse model due to the lack of functional FXR in that model. Nevertheless, it remains highly relevant to evaluate the impact of persistently elevated plasma BA levels on the heart of *Cyp2c70*^−/−^ mice.

In conclusion, we demonstrate that elevated plasma BA levels are associated with cardiac stress and inflammation in *Cyp2c70*^−/−^ pups with characteristics of cholestasis, which could be prevented by perinatal UDCA treatment. Numerous genes that have been linked to cardiovascular diseases were altered in hearts of hypercholanemic *Cyp2c70*^−/−^ pups and correlated to plasma BA levels. Altogether, these data reveal that exposure to high circulating BA concentrations profoundly impacts cardiac gene expression patterns, which might ultimately lead to myocardial dysfunction. Future, long-term, studies in mice with a human-like BA composition will however be required to further unravel the mechanisms by which BAs impact cardiac function.

## Supplementary information

Supplementary material

## Data Availability

The datasets generated during and/or analyzed during the current study are available from the corresponding author on reasonable request.
