# Supplementary material for: Elevated plasma bile acids coincide with cardiac stress and inflammation in young Cyp2c70−/− mice
Source: Pediatr Res. 2024 Oct 2;97(6):2145–52. doi: 10.1038/s41390-024-03596-4 (PMC12122362; doi:10.1038/s41390-024-03596-4)
Supplement: Supplementary file 1 — Supplementary material [file 41390_2024_3596_MOESM1_ESM.pdf]

**--- SUPPLEMENTARY MATERIAL ---**

# Elevated plasma bile acids coincide with cardiac stress and inflammation in young *Cyp2c70*$^{-/-}$ mice

Hilde D. de Vries[1,2,6], Tim R. Eijgenraam[3,4,6], Vincent W. Bloks[3], Niels L. Mulder[3], Tim van Zutphen[2,3], Herman H.W. Silljé[4], Folkert Kuipers[3,5,6*] and Jan Freark de Boer[1,3,6*]

[1]Department of Laboratory Medicine, University of Groningen, University Medical Center Groningen, Groningen, The Netherlands

[2]Faculty Campus Fryslân, University of Groningen, Leeuwarden, The Netherlands

[3]Department of Pediatrics, University of Groningen, University Medical Center Groningen, Groningen, The Netherlands

[4]Department of Cardiology, University of Groningen, University Medical Center Groningen, Groningen, The Netherlands

[5]European Research Institute for the Biology of Ageing (ERIBA), University of Groningen, University Medical Center Groningen, Groningen, The Netherlands

[6]These authors contributed equally: Hilde D. de Vries and Tim R. Eijgenraam; Folkert Kuipers and Jan Freark de Boer

## Supplementary Figures

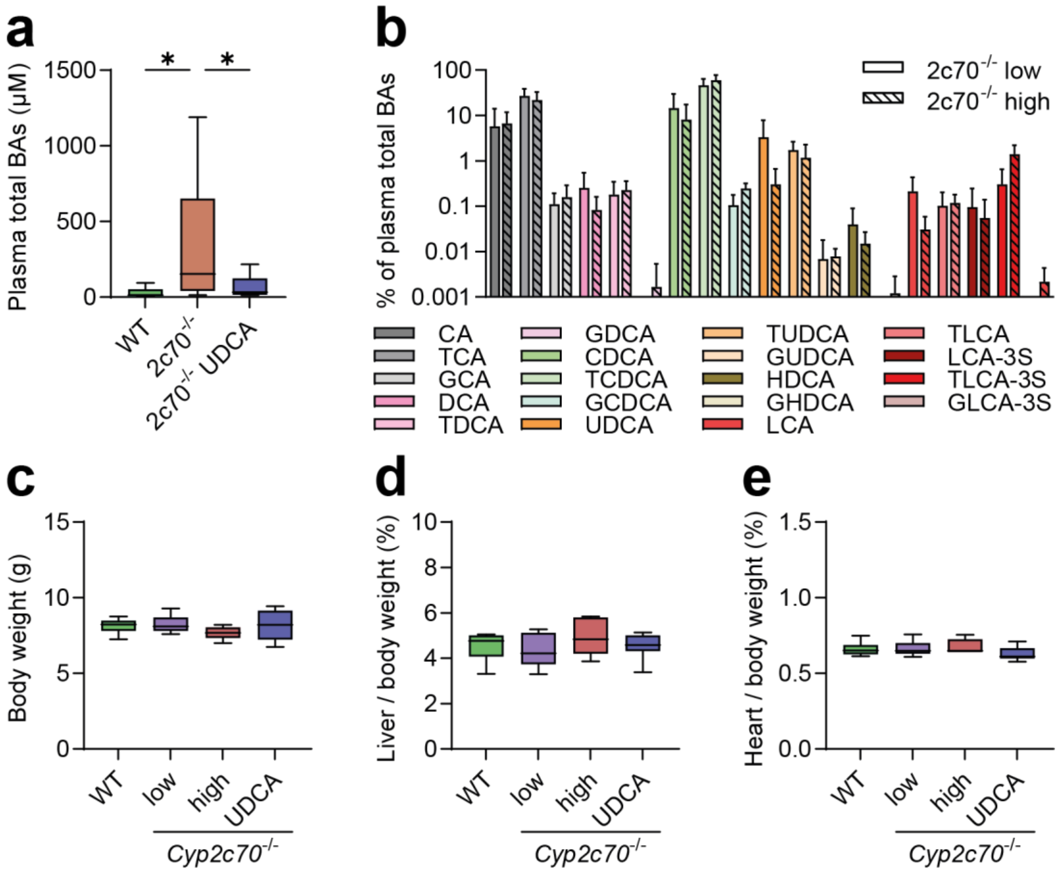


**Supplementary Fig. 1** **a** Plasma total BA levels of WT pups ($n$ = 6), *Cyp2c70*$^{-/-}$ pups ($n$ = 11) and *Cyp2c70*$^{-/-}$ pups of which the dams were perinatally treated with UDCA ($n$ = 12). *$P < 0.05$ (Kruskal-Wallis test followed by Conover post hoc comparisons). **b** Relative abundance of different BA species in plasma of *Cyp2c70*$^{-/-}$ pups with low or moderately elevated plasma BAs ('2c70$^{-/-}$ low', $n$ = 6) and *Cyp2c70*$^{-/-}$ pups with high plasma BAs ('2c70$^{-/-}$ high', $n$ = 5). Data are presented as means ± standard deviations (SD). Body weight (**c**) and liver (**d**) and heart (**e**) weight as percentage of body weight of WT pups ($n$ = 6), *Cyp2c70*$^{-/-}$ pups with low or moderately elevated plasma BAs ('2c70$^{-/-}$ low', $n$ = 6), *Cyp2c70*$^{-/-}$ pups with high plasma BAs ('2c70$^{-/-}$ high', $n$ = 5) and *Cyp2c70*$^{-/-}$ pups of which dams were perinatally given UDCA ($n$ = 12). 2c70, *Cyp2c70*; BA, bile acid; UDCA, ursodeoxycholic acid; WT, wild type.

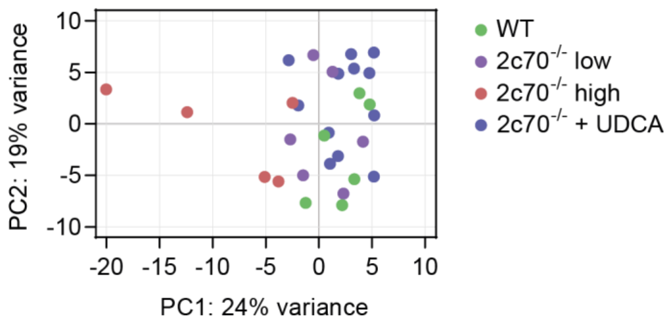


**Supplementary Fig. 2** PCA plot of cardiac gene expression profiles of WT pups ($n$ = 6), *Cyp2c70*$^{-/-}$ pups with low or moderately elevated plasma BAs ('2c70$^{-/-}$ low', $n$ = 6), *Cyp2c70*$^{-/-}$ pups with high plasma BAs ('2c70$^{-/-}$ high', $n$ = 5) and *Cyp2c70*$^{-/-}$ pups of which dams were perinatally administered UDCA ($n$ = 12).

## Supplementary Tables

**Supplementary Table 1.** Significantly enriched hallmark gene sets in differential expression between *Cyp2c70*$^{-/-}$ pups with high plasma BAs versus *Cyp2c70*$^{-/-}$ pups with low or moderately elevated plasma BAs.

| Pathway | Pval | Padj | ES | NES | Leading edge genes |
|---|---|---|---|---|---|
| Xenobiotic metabolism | 1.76e-07 | 8.79e-06 | 0.43 | 2.01 | *Gabarapl1, Ptgds, Hsd11b1, Il1r1, Tnfrsf1a, Xdh, Hmox1, Fas, Ahcyl, Acox1, Gcnt2, Por, Dcxr, Gclc, Man1a, Kars, Gnmt, Fah, Tmem176b, Comt, Nqo1, Gsto1, Ndrg2, Ninj1, Aox1, Acox3, Cyp2j6, Cd36, Pmm1, Cdo1, Tat, Papss2, Nmt1, Adh5, Cndp2, Gstm4, Slc6a6, Serpine1, Cyp27a1, Itih4, Pcx, Aldh9a1, Atoh8, Crot, Cyp1a1, Upp1, Marchf6, Dhps, Gss, Abcd2, Pros1, Pdk4, Tmbim6, Gart, Acp1, Retsat, Cat, Aco2, Pink1, Abcc3, Acp2, Vnn1, Maoa, Npc1, Asl, Lpin2, Slc1a5, Slc46a3, Aldh2, Apoe* |
| TNF-α signaling via NF-κB | 6.63e-07 | 1.66e-05 | 0.41 | 1.96 | *Pfkfb3, Socs3, Cebpd, Dusp4, Cdkn1a, Myc, Maff, Spsb1, Map3k8, Fjx1, Ccrl2, Klf9, Nfkbia, Junb, Abca1, Litaf, Birc3, Il6st, Nampt, Sqstm1, Slc16a6, Ppp1r15a, Gadd45b, Mcl1, Birc2, Mxd1, Btg3, Icam1, Tnip1, Sat1, Tnip2, Ninj1, Bcl3, Gfpt2, Tiparp, Ptgs2, Zfp36, Pnrc1, Klf6, Sphk1, Bmp2, Rnf19b, Snn, Sgk1, Ptger4, Icosl, Msc, Slc2a3, Serpine1, Nfkb2, Serpinb8, Plek* |
| Androgen response | 4.44e-05 | 2.77e-04 | 0.43 | 1.85 | *Fkbp5, Maf, Selenop, Arid5b, Map7, Sord, Ptk2b, Steap4, Iqgap2, Zmiz1, Ngly1, Pgm3, Tmem50a, Sat1, Camkk2, Pias1, Gpd1l, Xrcc5, Hpgd, Ndrg1, Mertk, Sgk1, Tpd52, Pa2g4, Herc3* |
| Hypoxia | 7.40e-06 | 7.40e-05 | 0.38 | 1.82 | *Pfkfb3, Cdkn1a, Foxo3, Maff, Mt1, Lox, Errfi1, Pdk1, Igfbp3, Pam, Klhl24, Hmox1, Rora, Cp, Cdkn1b, Dcn, Gcnt2, Tgm2, Ccn2, Hdlbp, Ppp1r15a, Cited2, Ddit4, Rragd, Pgf, Ccng2, Scarb1, Ppargc1a, Mxi1, Kdm3a, Tiparp, Vldlr, Zfp36, Plin2, Pnrc1, Ndrg1, Gaa, Klf6, Selenbp1, Ndst2, Tpd52, Ndst1, Egfr, Nedd4l, Adora2b, Slc2a3, Slc6a6, Serpine1, Adm, Atp7a, Slc2a1, Bnip3l* |
| IL6-JAK-STAT3 signaling | 2.75e-04 | 9.81e-04 | 0.44 | 1.81 | *Il4ra, Socs3, Osmr, Il1r1, Map3k8, Tnfrsf1a, Tnfrsf1b, Hmox1, Fas, Stat3, Ccr1, Ptpn11, Il6st, Cd14, Lepr, Stam2, Cd36, Ltbr, Ptpn2, Il17ra, Acvr1b, Ifngr1, Pik3r5, Csf3r, Ifnar1, Ptpn1, Myd88, Cntfr* |
| Bile acid metabolism | 2.63e-04 | 9.81e-04 | 0.42 | 1.78 | *Optn, Abca1, Lonp2, Gnmt, Pex12, Abca3, Idh2, Paox, Tfcp2l1, Rxra, Lipe, Abca6, Pxmp2, Ch25h, Nedd4, Hsd17b11, Aldh1a1, Cyp27a1, Gnpat, Aldh9a1, Slco1a4, Crot, Abca5, Bcar3, Abcd2, Scp2, Cyp39a1, Bmp6, Retsat, Abca9, Cat, Nr0b2, Pex7, Mlycd, Gclm, Npc1, Pnpla8* |
| Inflammatory response | 1.30e-04 | 6.52e-04 | 0.36 | 1.68 | *Il4ra, Osmr, Cdkn1a, Myc, Slc31a2, Il1r1, Tnfrsf1b, Ccrl2, Nfkbia, Abca1, Nampt, Rnf144b, Msr1, Mxd1, Icam1, Cd14, Sema4d, Cybb, C3ar1, Atp2c1, Nod2, Hpn, Ifitm1, Klf6, Sphk1, Hif1a, Ptger4, Icosl, Acvr1b, Adora2b, Met, Serpine1, Adm, P2rx4, Pik3r5, Tnfrsf9, Cmklr1, Irak2, Raf1, Plaur, Csf3r, Ptger2, Ifnar1, Slc4a4, Ptpre, Pvr, Ahr, Best1, Tnfaip6, Selenos, Ccl24, Ptafr, Abi1, Irf7, Adgre1, Itgb3, Stab1* |
| Estrogen response early | 2.53e-04 | 9.81e-04 | 0.35 | 1.65 | *Fkbp5, Syt12, Retreg1, Myc, Ugcg, Inhbb, Mindy1, Ablim1, Nrip1, Tgm2, Kdm4b, Il6st, Fdft1, Med13l, Xbp1, Jak2, Dhrs3, Igf1r, Sh3bp5, Muc1, Scarb1, Svil, Elf1, Abca3, Mybbp1a, Slc37a1, Tiparp, Mlph, Amfr, Gfra1, Tpd52l1, Aff1, Fasn, Papss2, Lad1, Adcy9, Foxc1, Slc2a1, Abat, Lrig1, B4galt1, Dlc1, Syngr1* |

| | | | | | |
|---|---|---|---|---|---|
| Heme metabolism | 3.32e-04 | 0.001 | 0.34 | 1.63 | *Tfdp2, Foxo3, Ezh1, Tns1, Osbp2, Cir1, Sptb, Bach1, Mospd1, Optn, Cast, Khnyn, Adipor1, Aldh6a1, Gclc, Abcg2, Nnt, Nek7, Epb41, Mxi1, Mocos, Slc6a8, Gde1, Fech, Kat2b, Dcaf11, Selenbp1, Xpo7, Slc30a1, Myl4, Gapvd1, Synj1, Klf3, Lamp2, Add1, Pcx, Slc2a1, Hebp1, Bnip3l, Marchf8, Ranbp10, Sdcbp, Sidt2, Riok3, Vezf1, Mgst3, Tnrc6b, Rhd, Ccdc28a, H1f0, Ctns, Cat, Gypc, Fbxo7, Foxj2, Gclm, Kdm7a, Lpin2, C3, Mkrn1, Marchf2, Slc25a38, Usp15, Rcl1* |
| IL2-STAT5 signaling | 5.89e-04 | 0.002 | 0.34 | 1.62 | *Il4ra, Tlr7, Plscr1, Gabarapl1, Cd86, Myc, Maff, Selp, Nt5e, Map3k8, Tnfrsf1b, Bcl2l1, Slc39a8, Ahcyl, Rora, Tgm2, Ptch1, Xbp1, Rragd, Dhrs3, Gadd45b, Igf1r, Muc1, Etfbkmt, Mxd1, Fah, Ttc39b, Prkch, Arl4a, Pth1r, Gsto1, Plin2, Ndrg1, Sh3bgrl2, Klf6, Smpdl3a, Bmp2, Bmpr2, Plagl1, Slc2a3, Ikzf4, Furin, Penk, Ifngr1, P2rx4, Tnfrsf9, Cyfip1, Lrig1, Abcb1a, Odc1, Ahnak, Batf, Ptger2, Tnfrsf18, Anxa4* |
| Protein secretion | 0.002 | 0.006 | 0.36 | 1.57 | *Bnip3, Ap3b1, Sgms1, Pam, Abca1, Golga4, Cog2, Mon2, Dop1a, Snx2, Dst, Tpd52, Copb2, Egfr, Gosr2, Mapk1, Lamp2, Ppt1, M6pr, Atp7a, Stx12, Arfgef2, Ctsc, Rab5a, Uso1, Napa, Atp6v1h, Ica1, Galc, Ap2b1, Tmed10, Tom1l1, Krt18, Arfgap3* |
| p53 pathway | 8.35e-04 | 0.003 | 0.32 | 1.53 | *Sesn1, Cdkn1a, Foxo3, Vwa5a, H1f2, Inhbb, Abhd4, Hmox1, Fas, Baiap2, Plxnb2, Mknk2, Slc3a2, Dcxr, Ppp1r15a, Tgfa, Ddit4, Alox8, Zbtb16, Xpc, Mxd1, Ppm1d, Ada, Sat1, Ninj1, Mxd4, Tpd52l1, Osgin1, Coq8a, Sp1, Ndrg1, Pmm1, Sphk1, Prkab1, Bmp2, Rnf19b, Dnttip2, Rxra, Fuca1, Acvr1b, Iscu, Pvt1, Ankra2, Rack1, Rb1, Abat, Tm7sf3, Txnip, Tchh, Trafd1, Upp1, Vdr, Apaf1, Ptpre, Tspyl2, Tprkb, Stom, Sec61a1, Ercc5, Retsat, Cdk5r1* |
| Complement | 0.005 | 0.011 | 0.32 | 1.49 | *Clu, Plscr1, Gng2, Maff, Pla2g7, Cfh, Cpm, Spock2, Ctsl, Cp, Pik3ca, Jak2, Mmp8, Serping1, Brpf3, Lipa, Casp4, Lap3, Ang, Cd36, Prdm4, Dusp6, Irf2, S100a9, Pla2g4a, Sh2b3, Serpine1, Cblb, Dgkg, Dock9, Ctss, Lamp2, Plek, Ctsh, Pik3r5, Casp9, Ctsc, Raf1, C1qc, Plaur, Ctso, Usp8, Pik3cg, Lrp1, Akap10, Cdk5r1, F8, Cebpb, Zfpm2, Lgals3, Irf7, Actn2, C1qa, C3, Notch4, Pclo, Usp15, Gp1ba, Cd46, Fcer1g, Ehd1, Lcp2, Adra2b, Gca, Pdp1* |
| Interferon gamma response | 0.002 | 0.005 | 0.31 | 1.47 | *Il4ra, Socs3, Plscr1, Cdkn1a, Cd86, Selp, Cfh, Nfkbia, Fas, Arid5b, Rnf31, Stat3, Trim25, Nampt, Jak2, Marchf1, Icam1, Serping1, Arl4a, Casp4, Lap3, Ptgs2, Ptpn2, Eif4e3, Hif1a, Peli1, Ogfr, Slc25a28, Irf2, Pla2g4a, Ripk1, Mvp, Cmklr1, Txnip, Rapgef6, Nod1, Trafd1, Upp1* |
| Apoptosis | 0.011 | 0.023 | 0.31 | 1.44 | *Clu, Gpx3, Cdkn1a, Bcl2l1, Hmox1, Fas, Timp3, Cdkn1b, Dcn, Hmgb2, Birc3, Sqstm1, Gadd45b, Mcl1, Rnasel, Avpr1a, Btg3, Tgfbr3, Cd14, Dpyd, Casp4, Sat1, Cth, Gsn, Hgf, Bmp2, Bmf, Psen2, Ifngr1, Ppt1, Add1, Casp9, Bnip3l, Casp2, Txnip, Ctnnb1, Crebbp, Brca1, H1f0, Retsat, Bcl2l11, Xiap, Casp8, Lgals3, Gstm2, Gucy2e, Cd38, Cyld, Krt18, Anxa1, Dnajc3, Rhob* |
| mTORC1 signaling | 0.002 | 0.004 | -0.29 | -1.49 | *Ddit3, Serpinh1, Egln3, Pfkl, Slc1a4, Cxcr4, Hspd1, Mllt11, Mcm2, Srd5a1, Nfyc, Tm7sf2, Phgdh, Dhcr24, Hmbs, Stip1, Dhcr7, P4ha1, Ak4, Hmgcs1, Hspe1, Ccnf, Ldlr, Fkbp2, Cyb5b, Cdc25a, Mthfd2, Tuba4a, Prdx1, Psph, Qdpr, Tubg1, Sqle, Glrx, Psme3, Hspa4, Atp5g1, Add3, Tomm40, Pik3r3, Hmgcr, Sec11a, Actr3, Eef1e1, Pgk1, Psmb5, Nupr1, Cacybp, Plk1, Ddx39a, Fdxr, Stard4, Nfil3, Cct6a, Hk2, Me1, Ifi30, Ssr1* |

| | | | | | |
|---|---|---|---|---|---|
| Apical junction | 2.62e-04 | 9.81e-04 | -0.32 | -1.63 | *Bmp1, Cercam, Cd34, Fscn1, Thy1, Jam3, Mdk, Mmp2, Col16a1, Actb, Mapk14, Actg1, Ywhah, Tnfrsf11b, Gamt, Cnn2, Parva, Stx4a, Pfn1, Myh10, Vasp, Cd276, Rhof, Actc1, Icam2, Thbs3, Nlgn3, Mpzl1, Zyx, Tubg1, Ppp2r2c, Nlgn2, Epb41l2, Ctnna1, Ptk2, Kcnh2, Speg, Tial1, Pik3r3, Itga9, Negr1, Myl12b, Rsu1, Evl, Itga2, Amh, Myl9, Inppl1, Col9a1, Itga10, Nectin3, Pbx2, Ctnnd1, Taok2, Adam23* |
| G2M checkpoint | 5.73e-04 | 3.18e-04 | -0.33 | -1.71 | *Atf5, Cks1b, Mapk14, Marcks, Pola2, Tacc3, Ube2c, Pbk, H2az1, Mcm2, Cdk1, Ccnd1, Nolc1, Cdk4, Hmgb3, Srsf2, Dkc1, Meis1, Ccnf, Kif4, Ndc80, Cdc25a, Cbx1, H2ax, Fancc, Birc5, Lmnb1, Katna1, Sqle, Nusap1, Tra2b, Ss18, Jpt1, Srsf1, Prc1, Nasp, Ccna2, E2f1, Nup50, Snrpd1, Kif22, Plk1, Cenpf, Ezh2, Ddx39a, E2f2, Mcm5, Kif23, Top2a, Ube2s, Cdc6, Gins2, Troap, Cdc7, Hnrnpd, Sap30, Cenpa, Hira, Nek2, Amd1, Hspa8, Upf1, Tfdp1, Incenp, Smad3, G3bp1, Ttk, Tgfb1, Pafah1b1, Racgap1* |
| Oxidative phosphorylation | 3.39e-05 | 2.42e-04 | -0.34 | -1.75 | *Ndufb2, Timm9, Ndufb3, Timm13, Cox6a1, Ndufc2, Mrpl34, Mrpl35, Tcirg1, Ndufab1, Ndufs4, Ndufs6, Mrps11, Ndufc1, AK157302, Atp5d, Cox8a, Dlst, Atp6ap1, Tomm22, Ndufb7, Ndufb6, Sdhc, Ndufa8, Ndufa3, Ndufa9, Uqcr11, Gm10053, Ndufb8, Atp5g1, Suclg1, Timm17a, Timm10, Ndufv2, Uqcrfs1, Etfb, Cyc1, Ndufb5, Fdx1, Mdh2, Ndufa1, Ndufa5, Vdac3, Polr2f, Atp5j, Hccs, Pdhb, Cox6b1, Ndufa2, Atp5k, Mrps12, Atp5e, Mdh1, Bax, Cox6c, Atp5o, Atp5g3, Uqcrq, Dlat, Cox5b, Slc25a11, Rhot2, Cox7a2, Ndufs8, Atp5j2, Atp5h, Ndufs3, Alas1, Atp6v1g1, Mtx2, Ndufs2, Vdac2, Cox5a, Idh3a, Cox11, Cox7c, Ndufs7, Uqcrh, Cox17, Hspa9, Idh3g, Sdhd, Grpel1, Slc25a5, Uqcrc1* |
| MYC targets V1 | 1.00e-05 | 8.34e-05 | -0.34 | -1.76 | *Erh, Hdgf, Lsm2, Ncbp2, Txnl4a, Glo1, Snrpd3, Ranbp1, Rps10, Dut, Hspd1, H2az1, Hnrnpa2b1, Mcm2, Fbl, Nolc1, Tardbp, Cdk4, Tufm, Srsf2, Ndufab1, Snrpa, Ywhaq, Srm, Hspe1, Trim28, Pcbp1, Nop56, Srsf7, Ncbp1, Cct3, Psmb2, Hnrnpa3, Tra2b, Gm10146, Srsf1, Ctps, Phb, Ccna2, Srsf3, Nop16, Ran, Snrpd1, Ruvbl2, Ssbp1, Pgk1, Cyc1, Rps2, Vdac3, Exosc7, Mcm5, Cct5, C1qbp, Hdac2, Ilf2, Eif4a1, Nme1, Hnrnpc, Tyms, Psmb3, Hnrnpd, Gm4705* |
| E2F targets | 5.87e-06 | 7.34e-05 | -0.35 | -1.79 | *Cks1b, Chek2, Pop7, Pola2, Tacc3, Ncapd2, Trp53, Ranbp1, Dut, Brms1l, H2az1, Mcm2, Cdk1, Nolc1, Psmc3ip, Depdc1a, Phf5a, Cdk4, Hmgb3, Rpa3, Snrpb, Srsf2, Ppp1r8, Tubb5, Pnn, Kif4, Cnot9, Nop56, Cdc25a, Mthfd2, H2ax, Birc5, Lmnb1, Tubg1, Slbp, Melk, Tra2b, Jpt1, Srsf1, Ctps, Pole4, Wdr90, Nasp, Luc7l3, Tbrg4, Ran, Rad51ap1, Kif22, Cdca3, Plk1, Exosc8, Ezh2, Ddx39a, Cdca8, Mcm5, Wee1, Asf1b, Top2a, Ube2s, Nme1, Hells, Rad1, Hnrnpd, E2f8, Mlh1, Diaph3, Tcf19, Dlgap5, Ccp110, Eed* |
| Angiogenesis | 0.002 | 0.005 | -0.52 | -1.88 | *Col3a1, Postn, Lum, Pdgfa, Msx1, Vtn, Fstl1, Ccnd2, Ptk2, Col5a2, Vegfa* |
| Epithelial mesenchymal transition | 1.39e-06 | 2.32e-05 | -0.37 | -1.88 | *Col6a2, Col3a1, Bmp1, Postn, Mest, Pcolce, Thy1, Col1a2, Tnc, Lgals1, Matn2, Fap, Col5a1, Mmp2, Col16a1, Lum, Serpinh1, Htra1, Col1a1, Loxl1, Vim, Anpep, Msx1, Acta2, Pmepa1, Efemp2, Nnmt, Col6a3, Tnfrsf11b, Sparc, Fstl1, Il15, Snai2, Vegfc, Fn1, Pfn2, Wnt5a, Jun, Col5a3, Bgn, Ecm1, Basp1, Plod1, Tpm4, Sdc1, Lrrc15, Adam12, Ppib, Magee1, Mfap5, Col5a2, Rgs4, Oxtr, Vegfa, Lamc1, Emp3, Tpm2, Itga5, Itga2, Eln, Ntm, Myl9, Nid2, Col7a1, Serpine2* |

**Supplementary Table 2.** Top ten significantly positively and negatively enriched Reactome pathways among differential expression between *Cyp2c70*$^{-/-}$ pups with high plasma BAs versus *Cyp2c70*$^{-/-}$ pups with low or moderately elevated plasma BAs.

| Pathway | Pval | Padj | ES | NES | Leading edge genes |
|---|---|---|---|---|---|
| Interleukin 6 family signaling | 1.93e-04 | 0.005 | 0.70 | 2.10 | *Socs3, Osmr, Il6ra, Stat3, Ptpn11, Il6st, Jak2* |
| Synthesis of PIPs at the early endosome membrane | 3.61e-04 | 0.007 | 0.70 | 2.01 | *Pi4k2b, Pi4k2a, Pik3c3, Mtmr2, Mtmr4, Mtm1, Mtmr12, Pik3r4, Inpp4b, Pikfyve, Pik3c2a, Fig4* |
| PI metabolism | 3.11e-05 | 9.07e-04 | 0.48 | 1.99 | *Ptpn13, Pi4k2b, Mtmr14, Pi4k2a, Pip4k2c, Pik3ca, Mtmr3, Pik3c3, Inpp5d, Pik3r1, Mtmr7, Mtmr6, Mtmr2, Gde1, Plekha6, Pip4p1, Pitpnb, Inpp5k, Synj1, Pip4k2a, Pik3r5, Mtmr1, Mtmr4, Rab5a, Mtmr9, Mtm1, Pik3cg, Mtmr12, Pip5k1a, Pik3r4, Inpp4b, Pikfyve, Pik3c2a* |
| Branched chain amino acid catabolism | 0.001 | 0.017 | 0.63 | 1.97 | *Aldh6a1, Acadsb, Ppm1k, Bckdha, Hsd17b10, Ivd, Hibadh, Echs1, Bckdhb, Auh, Dbt* |
| Antimicrobial peptides | 0.001 | 0.017 | 0.65 | 1.96 | *Lcn2, Clu, Lyz2, Art1, S100a8, S100a9, Slc11a1, Atp7a* |
| Synthesis of PIPs at the plasma membrane | 3.67e-04 | 0.007 | 0.50 | 1.89 | *Ptpn13, Pi4k2b, Mtmr14, Pi4k2a, Pip4k2c, Pik3ca, Mtmr3, Inpp5d, Pik3r1, Mtmr6, Plekha6, Inpp5k, Synj1, Pip4k2a, Pik3r5, Mtmr1, Rab5a, Mtmr9, Mtm1, Pik3cg, Pip5k1a, Inpp4b* |
| TBC RabGAPs | 9.24e-04 | 0.014 | 0.53 | 1.86 | *Tbc1d15, Map1lc3b, Optn, Tsc1, Ulk1, Tbc1d17, Tsc2, Rabgap1, Rab7b, Tbc1d14, Tbc1d24, Rab7, Gabarap, Tbc1d25* |
| Interleukin 1 family signaling | 2.38e-04 | 0.005 | 0.40 | 1.79 | *Irak3, Il1r1, Map3k8, Nfkbia, Stat3, Psmd11, Sqstm1, Traf6, Map3k3, Tnip2, Nkiras2, Tollip, Tifa, Psmd2, Il33, Ubb, Psmd6, Peli1, Psmd1, Psmc1, Peli2, Nfkbib, Alpk1, Irak4, Nfkb2, Psmd4, Psmf1, Irak2, Cul1, Tbk1, Myd88, Tab1, Psmd7, Psma6, Psme4, Casp8, Psmd13, Psma7, Il1rn* |
| HATs acetylate histones | 0.001 | 0.016 | 0.49 | 1.79 | *Jade2, Kansl3, Brpf3, Phf20, Ncoa2, Ing5, Kansl1, H2bc4, H3c6, Kat6b, Atf2, Jade1, Brpf1, H2bc8, Mcrs1, Brd1, Kat6a* |
| Sphingolipid metabolism | 0.002 | 0.020 | 0.43 | 1.74 | *Acer2, Sgms1, Spns2, Ugcg, Sgpl1, Sptlc2, Esyt2, Neu3, Sphk1, Hexb, B3galnt1, Kdsr, Sptlc1, Arsg, M6pr, Asah2, Cerk, Esyt1, Aldh3b3, Arsk, Ctsa, Sphk2, Smpd3, Galc, Gba2* |
| Mitochondrial translation | 5.68e-11 | 5.67e-09 | -0.56 | -2.52 | *Mrps36, Mrpl51, Mrpl49, Mrps17, Mrpl34, Mrpl13, Mrpl18, Mrpl35, Mrps21, Mrpl37, Mrps11, Mrpl28, Mrpl55, Mrpl48, Mrps33, Mrps6, Mrpl21, Mrps14, Mrpl12, Mrpl36, Mrpl33, Mrps9, Mrpl22, Mrpl46, Mrpl19, Mrps25, Mrps34, Mrps28, Mrpl42, Mrpl47, Mrpl10, Mrpl45, Gfm1, Mrpl27, Mrps12, Mrpl16, Mrpl4, Chchd1, Mrps16, Mrpl38, Mrpl14, Mtrf1l, Mrpl41, Mrpl39, Mrpl52, Mrpl1, Mrps10, Mrpl32, Mrpl54, Mrps7, Mrpl20, Ptcd3, Mrpl50, Mrpl23, Mrpl2, Mrps22, Mrps23, Mrps27, Mrps2, Mrps18c, Mrps26, Mrrf, Mrpl57, Mrps15, Mrps24, Oxa1l, Mrps18a, Mrpl15, Gfm2, Mrpl3, Aurkaip1, Gadd45gip1* |
| Integrin cell surface interactions | 6.97e-10 | 4.87e-08 | -0.59 | -2.52 | *Col6a2, Col3a1, Col6a1, Col1a2, Tnc, Jam3, Col5a1, Col16a1, Col6a6, Lum, Itgb6, Col1a1, Col6a3, Vtn, Col4a4, Col9a3, Icam2, Fn1, Col5a3, Itgax, Itga2b, Col4a6, Col18a1, Col8a1, Col2a1, Col5a2, Itga9, Itga5, Itga8, Itga2, Col9a1, Itga10, Itgb7, Col7a1* |
| Extracellular matrix organization | 2.52e-17 | 1.76e-14 | -0.50 | -2.58 | *Mfap4, Col6a2, Col3a1, Bmp1, Col6a1, Col26a1, Pxdn, Pcolce, Col1a2, Matn4, Tnc, Jam3, Col5a1, Col27a1, Ltbp4, Mmp2, P3h3, Col16a1, Col6a6, Lum, Itgb6, Serpinh1, Htra1, Col1a1, Loxl1, Col14a1, Lama4, Tgfb2, Col15a1, Efemp2, Col6a3, Mfap2, Sparc, P4ha2, Vtn, Col4a4,* |

| | | | | | |
|---|---|---|---|---|---|
| | | | | | *Loxl2, Col22a1, Colgalt2, Emilin1, Col9a3, Emilin2, Icam2, Itga6, Fn1, Mmp15, P4ha1, Scube1, Ctsk, Capn5, Col5a3, Bgn, Agrn, Tgfb3, Adam8, Itgax, Tnxb, Itga2b, Tnr, Capn15, Crtap, Col4a6, Tll2, Col18a1, Plod1, Sdc1, Adam12, Col8a1, Ppib, Mmp25, Adamts14, Col2a1, Timp2, Col11a2, Mfap5, Col5a2, Tpsb2, Itga9, Itga7, Itga5, Itga8, Itga2, Eln, Col9a1, Itga10, Itgb7, Col24a1, Nid2, Capn3, Col7a1, Col20a1, Fbn1, Ddr2, P3h1, Ltbp2, Ltbp1, Col4a2, Col25a1, Cma1* |
| Complex I biogenesis | 1.31e-10 | 1.14e-08 | -0.64 | -2.64 | *Ndufb2, Ndufb3, Ndufv3, Ndufaf1, Ndufb10, Ndufc2, mt-Nd3, Ndufab1, Ndufs5, Ndufs4, Ndufs6, Ndufaf2, Ndufc1, Ndufa11, Ndufb7, Ndufb6, Ndufa8, Ndufa3, Ndufa9, Ndufa12, Ndufaf4, Ndufb8, Ndufv2, Ndufaf6, Ndufb5, Ndufa1, Ndufa5, Ndufa2, Ndufa13, Ndufs8, Ndufb4, Ndufaf3, Tmem186, Ndufs3, Ndufaf5, Tmem126b, Ndufs2, Timmdc1, Ndufs7, Ndufb9, Ndufa10* |
| Collagen formation | 1.43e-11 | 2.00e-09 | -0.60 | -2.64 | *Col6a2, Col3a1, Bmp1, Col6a1, Col26a1, Pxdn, Pcolce, Col1a2, Col5a1, Col27a1, P3h3, Col16a1, Col6a6, Serpinh1, Col1a1, Loxl1, Col14a1, Col15a1, Col6a3, P4ha2, Col4a4, Loxl2, Col22a1, Colgalt2, Col9a3, P4ha1, Col5a3, Crtap, Col4a6, Tll2, Col18a1, Plod1, Col8a1, Ppib, Adamts14, Col2a1, Col11a2, Col5a2, Col9a1, Col24a1, Col7a1, Col20a1, P3h1, Col4a2, Col25a1, Ctsb* |
| Respiratory electron transport ATP synthesis | 1.38e-14 | 3.81e-12 | -0.56 | -2.68 | *Ndufb2, Cox19, Ndufb3, Ndufv3, Ndufaf1, Ndufb10, Cox14, Cox6a1, Ndufc2, Cycs, mt-Nd3, Ndufab1, Ndufs5, Ndufs4, Ndufs6, Ndufaf2, Ndufc1, Atp5d, Ndufa11, Cox8a, Ndufb7, Ndufb6, Ndufa8, Ndufa3, Ndufa9, Uqcr11, Gm10053, Ndufa12, Ndufaf4, Ndufb8, Atp5g1, Sco1, Ndufv2, Uqcrfs1, Etfb, Cyc1, Ndufaf6, Ndufb5, Cox20, Ndufa1, Ndufa5, Cox18, Atp5j, Cox6b1, Ndufa2, Ndufa13, Atp5k, Taco1, Atp5e, Atp5o, Atp5g3, Uqcrq, Cox5b, Ndufs8, Ndufb4, Ndufaf3, Atp5j2, Tmem186, Atp5h, Ndufs3, Coq10b, Ndufaf5, Tmem126b, Ndufs2, Cox5a, Cox11, Timmdc1, Cox7c, Ndufs7, Cox16, Uqcrh, Ndufb9, Ndufa10, Atp5l* |
| Collagen chain trimerization | 2.98e-10 | 2.31e-08 | -0.73 | -2.73 | *Col6a2, Col3a1, Col6a1, Col26a1, Col1a2, Col5a1, Col27a1, Col16a1, Col6a6, Col1a1, Col14a1, Col15a1, Col6a3, Col22a1, Col9a3, Col5a3, Col18a1, Col8a1, Col2a1, Col11a2, Col5a2, Col9a1, Col24a1, Col7a1, Col20a1* |
| Respiratory electron transport | 6.05e-14 | 1.06e-11 | -0.60 | -2.74 | *Ndufb2, Cox19, Ndufb3, Ndufv3, Ndufaf1, Ndufb10, Cox14, Cox6a1, Ndufc2, Cycs, mt-Nd3, Ndufab1, Ndufs5, Ndufs4, Ndufs6, Ndufaf2, Ndufc1, Ndufa11, Cox8a, Ndufb7, Ndufb6, Ndufa8, Ndufa3, Ndufa9, Uqcr11, Gm10053, Ndufa12, Ndufaf4, Ndufb8, Sco1, Ndufv2, Uqcrfs1, Etfb, Cyc1, Ndufaf6, Ndufb5, Cox20, Ndufa1, Ndufa5, Cox18, Cox6b1, Ndufa2, Ndufa13, Taco1, Uqcrq, Cox5b, Ndufs8, Ndufb4, Ndufaf3, Tmem186, Ndufs3, Coq10b, Ndufaf5, Tmem126b, Ndufs2, Cox5a, Cox11, Timmdc1, Cox7c, Ndufs7, Cox16, Uqcrh, Ndufb9, Ndufa10* |
| ECM proteoglycans | 4.78e-11 | 5.57e-09 | -0.70 | -2.77 | *Col6a2, Col3a1, Col6a1, Col1a2, Matn4, Tnc, Col5a1, Col6a6, Itgb6, Col1a1, Col6a3, Sparc, Vtn, Col9a3, Fn1, Col5a3, Bgn, Agrn, Itgax, Tnxb, Itga2b, Tnr, Col2a1, Col5a2, Itga9, Itga7, Itga8, Itga2, Col9a1* |
| Collagen biosynthesis and modifying enzymes | 1.63e-14 | 3.81e-12 | -0.68 | -2.88 | *Col6a2, Col3a1, Bmp1, Col6a1, Col26a1, Pcolce, Col1a2, Col5a1, Col27a1, P3h3, Col16a1, Col6a6, Serpinh1, Col1a1, Col14a1, Col15a1, Col6a3, P4ha2, Col4a4, Col22a1, Colgalt2, Col9a3, P4ha1, Col5a3, Crtap, Col4a6, Tll2, Col18a1, Plod1, Col8a1, Ppib, Adamts14, Col2a1, Col11a2, Col5a2, Col9a1, Col24a1, Col7a1, Col20a1, P3h1, Col4a2, Col25a1* |

**Supplementary Table 3.** Description of the genes that are displayed in Fig. 4b-g.

| **Gene symbol** | **Name** | **Description** |
|---|---|---|
| *Serpina3n* | Serine (or cysteine) peptidase inhibitor, clade A, member 3N | A serine protease inhibitor, contributing to various physiological and pathological processes, including inflammation, immune response, tissue remodeling and potentially neuroprotection. |
| *Csf2rb2* | Colony stimulating factor 2 receptor, beta 2, low-affinity | A common beta subunit that forms part of the receptor complex for CSF, IL-3 and IL-5. These receptors play essential roles in proliferation, survival, differentiation, hematopoiesis and immune regulation. |
| *Cyp1b1* | Cytochrome P450, family 1, subfamily b, polypeptide 1 | An enzyme involved in the metabolism of various substances, including endogenous compounds (including steroids and fatty acids), xenobiotics (such as drugs and toxins) and carcinogens. |
| *Mycn* | N-myc proto-oncogene protein | A proto-oncogene that encodes a transcription factor involved in regulating cell proliferation, growth, and differentiation. Plays a crucial role in normal (embryonic) development but is also implicated in the pathogenesis of several cancers, especially neuroblastoma. |
| *Nrep* | Neuronal regeneration related protein | A protein involved in neuronal regeneration, cell migration, and differentiation. Aids in neurite outgrowth and repair after injury. Interacts with the TGF-β signaling pathway, influencing various cellular processes. |
| *Prr33* | Proline rich 33 | A protein belonging to the family of proline-rich proteins, which are known for their roles in protein-protein interactions and involvement in various cellular processes. While the specific functions and clinical significance of PRR33 are not well-documented, it is likely involved in cellular signaling and structural functions due to the properties of proline-rich domains. |
